# First language translation involvement in second language word processing

**DOI:** 10.3389/fpsyg.2022.986450

**Published:** 2022-09-08

**Authors:** Tao Zeng, Chen Chen, Jiashu Guo

**Affiliations:** School of Foreign Languages, Hunan University, Changsha, China

**Keywords:** second language, first language, translation, language proficiency, word processing

## Abstract

Studies on bilingual word processing have demonstrated that the two languages in a mental lexicon can be parallelly activated. However, it is under discussion whether the activated, non-target language gets involved in the target language. The present study aimed to investigate the role of the first language (L1, the non-target one) translation in the second language (L2, the target one) word processing. The tasks of semantic relatedness judgment and lexical decision were both adopted, to explore the relation of the possible L1 involvement and the task demand. Besides, bilinguals with relatively higher and lower L2 proficiency were recruited, to clarify the potential influence of L2 proficiency. Results showed that the manipulation of L1 translation exerted an influence on bilinguals’ task performances, indicating that L1 translation was involved, but did not just serve as a by-product when bilinguals were processing L2 words. And about the influence of L2 proficiency, the higher proficiency bilinguals performed better than the lower proficiency ones when the L1 translations could be taken advantage of, indicating a better access to L1 translation in L2 word processing, as bilinguals’ L2 proficiency increased. As for the task demands, the L1 translation was partially involved in Experiment 1 while a full involvement was observed in Experiment 2, suggesting a differed depth of L1 translation involvement, if the task demands allowed. The present study supplemented the previous ones due to its participants (the intermediate bilinguals) and tasks (the tasks of semantic relatedness judgment and lexical decision); besides, it provided an interesting view into interpreting the “task schema” of the BIA+ model.

## Introduction

A core issue in research on bilingualism is how bilinguals access words in their two languages. Numerous studies have been conducted accordingly, proposing two competing views. Early research suggested the language-selective view, an idea that bilinguals selectively activate words from only the target language (see, e.g., Watkins and Peynrcoğlu, 1983; Scarborough et al., 1984; Gerard and Scarborough, 1989). The non-selective view, on the other hand, holds that words from both the target and the non-target languages are activated (see, e.g., Beauvillain and Grainger, 1987; Green, 1998; Meuter and Allport, 1999). In recent decades, there has been a bulk of studies supporting the non-selective standpoint: it is claimed that words in the non-target language are also activated, even when bilinguals are only engaged in the target one. Corresponding evidence comes from experiments on homographs (Schwartz and Kroll, 2006), cognates (Van Hell and de Groot, 2008; Van Assche et al., 2009), semantically related words (Kroll et al., 2008), etc. There are also some theoretical models of bilingual lexical processing which defend the non-selective view, such as the IC model (Inhibitory Control Model, Green, 1998), BIA+ model (Bilingual Interactive Activation Model+, Dijkstra and Van Heuven, 2002; Dijkstra, 2005), and The Three-Stage Model of L2 Lexical Development (Jiang, 2000).

Despite the compelling evidence for non-selective activation, the role of the non-target language is still being discussed: does the activated non-target language act as a by-product (it is only activated), or does it get involved in the target language processing (it is activated, meanwhile helps to process the target language)? Several experiments were therefore conducted, with a special interest in the role of the first language (L1, the non-target one) during the second language (L2, the target one) processing. The participation of L1 in L2 processing was therefore revealed: the psychological experience of L1 was automatically activated in L2 tasks (e.g., Vukovic and Williams, 2014; Ahlberg et al., 2017); the neural networks of L1 orthographic, semantic and phonetic processing were active in L2 tasks (e.g., Tan et al., 2003; Xue et al., 2004; Nelson et al., 2009; Gao et al., 2015); the L1 knowledge on lexical meaning and collocation played a role when one dealt with L2 words (e.g., Wolter and Gyllstad, 2011; Zhang et al., 2017; Cao, 2018). To be noticed, the above studies focused on the involvement of L1 language, e.g., the processing strategy and knowledge that take a part in L2 use. Such L1 language involvement is at a much more general scale, possibly with no access to a certain L1 word. We thus turn to the specific aspect, that is, whether the L1 translation equivalents get involved in L2 use.

Some prior studies have been conducted to resolve the issue, adopting the paradigms of cross-language priming (Beauvillain and Grainger, 1987; Gerard and Scarborough, 1989; Dijkstra et al., 2000) or word translation (De Groot and Hoeks, 1995; Dufour and Kroll, 1995). The above paradigms took L2 word as the prime; the L1 translation was also presented as the target or spoken out as the response, which requires the obligate processing of both L2 and L1. Therefore, it is difficult to tell whether the possible L1 translation involvement is due to the L1 mediation in L2 word processing, or just due to the task requirement (Grosjean, 1998). For that reason, recent studies switched to monolingual tasks, within which the experimental stimuli and the required responses are only associated with L2 words. It is in such a manner that the obligatory processing of L1 can be avoided. The recently employed tasks are semantic relatedness judgment (e.g., Thierry and Wu, 2004, 2007) or lexical decision (e.g., Jiang et al., 2019). In both tasks, participants are asked to accomplish L2 tasks. Unknown to them, the words’ L1 translations are manipulated: in the semantic relatedness judgment task, the L1 translations of an L2 word-pair could share a logo-graphic character or not (translation repetition/non-repetition); and in the lexical decision task, the L1 translation of a word has a high or low lexical frequency (high/low translation frequency). Supposing that, in either task, the manipulated L1 translation leads to participants’ differing performances, the L1 translation involvement in L2 word processing can thus be verified.

The above two tasks, however, might be different in nature. In the lexical decision task, there exists a full activation of the L1 translation word, due to the manipulation of its word frequency. But as for the task of semantic relatedness judgment, it has been proposed that the manipulation of a shared character in L1 translation, essentially, creates a kind of L1 form-repetition. It was the repetition of a character that helped to promote L2 word processing, without the need to activate a whole L1 translation word (Costa et al., 2017; Jiang et al., 2019). And in that case, the L1 translation word may get fully involved in the lexical decision task, but partially involved in the task of semantic relatedness judgment. It is therefore worth exploring, whether the L1 translation involvement, if it exists, varies in depth due to the different task demands? To tackle this issue, both the task of semantic relatedness judgment and lexical decision will be adopted in the present study to test the same participants. If the L1 translation is involved in both tasks, the varied depth of L1 translation involvement on different task demands can be demonstrated.

Additionally, L2 proficiency is another factor that can affect the potential L1 translation involvement. Some previous studies agreed on the involvement of L1 translation, but with the disagreement on how the bilingual’s L2 proficiency plays a part. Li et al. (2018) analyzed high- and low-proficient Chinese-English bilinguals’ responses in the task of semantic relatedness judgment. They found that while the target words were semantically related, the effect of L1 activation (measured in reaction times) was greater in high proficiency group, than in its low proficiency counterpart. It indicated an increasing involvement of L1 translation with the L2 promotion. Hu and Qi (2014) reached the opposite conclusion, however. In their experiment, high- and low-proficient Chinese-English bilinguals performed a lexical decision task. The high proficiency group showed no different reaction times for the target English words (whose Chinese translations were acquired in advance) and the non-target ones (with no such acquisition), yet the significantly shorter reaction times for target English words were observed in the low-proficient group. The contrast suggested a decrease in L1 translation involvement as L2 proficiency was promoted. As shown above, results of the previous studies are not consistent. The present study will thus recruit the higher- and lower- proficient bilinguals; once the two proficiency groups are differed in task performances, the influence of L2 proficiency can be revealed.

In considering all those facts, the present study attempts to investigate the role of L1 translation in L2 word processing. Both the tasks of semantic relatedness judgment and lexical decision are adopted, with the consideration of a possible influence of task demand. Besides, bilinguals of higher- and lower- L2 proficiency are recruited. The two groups’ task performances are to be compared, through which we may investigate how bilingual’s L2 proficiency affects the possibly involved L1 translation in L2 word processing.

Accordingly, the research questions are stated as: (1) What is the role of the L1 translation in L2 word processing (get involved, or serve as a by-product)? (2) If the L1 translation gets involved in L2 word processing, what is the influence of L2 proficiency and task demands?

## Experiment 1: Semantic relatedness judgment

### Method

#### Participants

Fifty-eight bilinguals aging averagely around 21.3 years of age from a major university in Hunan participated in the experiment. All participants are right-handed with normal or corrected to normal eyesight. They are all native Chinese speakers who learn English as their second language, without experience of living or studying abroad. The participant’s L2 proficiency level is measured by their scores on the Test for English Majors-Band 8 (TEM-8, an English proficiency test for English majors in China; the passing of the test indicates a relatively high level of proficiency) and College English Test-Band 4 (CET-4, an English proficiency test for Chinese college students). Participants were asked to complete the Oxford Quick Placement Test (OQPT), grouped according to their test score, as well as L2 proficiency level and learning duration of English: the higher proficiency group, consisting of 29 participants (mean age = 23.1, OQPT = 48.5/60, learning duration = 13 years and 7 months, with a proficiency level not lower than TEM-8 or a CET-4 score higher than 600/710); and the lower proficiency group, consisting of 29 participants (mean age = 19.5, OQPT = 31.1/60, learning duration = 10 years and 7 months, with a proficiency level not higher than CET-4 or a CET-4 score lower than 400/710).

#### Materials

Forty English word pairs (eighty words) were selected from the British National Corpus (BNC) as the target stimuli. The words in a pair were matched in length and frequency. In each pair, the words were either semantically related (e.g., college-student) or unrelated (e.g., sport-fork). And unknown to participants, the Chinese translations of the word pair may share a Chinese character (e.g., college-student, translated as 学院-学生) or not (e.g., sport-fork, translated as 运动-叉子) (the repeated character, if any, was of the same position in their Chinese translations). Four conditions were therefore created: related & repeated, unrelated & repeated, related & unrepeated, and unrelated & unrepeated (see Table 1).

**TABLE 1:** Conditions (semantic relatedness and repetition in translation of the word pair) in the task of semantic relatedness judgment.

Repetition in translation (Implicit factor)	Semantic relatedness (Explicit factor)	
	
	Semantic related (S+)	Semantic unrelated (S−)
Translation repeated (T+)	**College—Student** 学院—学生 SRE 4.82 (0.13) SRC 4.81 (0.17)	**Angel—Genius** 天使—天才 SRE 1.49 (0.46) SRC 1.54 (0.49)
Translation unrepeated (T−)	**Milk—Bread** 牛奶—面包 SRE 4.35 (0.41) SRC 4.03 (0.52)	**Sport—Fork** 运动—叉子 SRE 1.34 (0.37) SRC 1.34 (0.46)

(1) A group of 10 high-level bilinguals (of Chinese and English), with no participation in the present study, was required to measure the mean semantic relatedness of the English stimuli (SRE, measured on a scale of 1–5), and the mean semantic relatedness of the stimuli translated into Chinese (SRC, measured on a scale of 1–5). (2) Standard deviations of SRE and SRC scores are given in parentheses. (3) The factor of Semantic Relatedness and Repetition in Translation were not correlated (SRE and Repetition in Translation: r = 0.156, p = 0.337; SRC and Repetition in Translation: r = 0.095, p = 0.560).

#### Procedure

After giving informed consent, participants were tested individually. They were instructed to perform the semantic relatedness judgment as fast and accurately as possible. Stimuli of the judgment were all presented visually, using the E-Prime 2.0 software (Psychology Software Tools, Pittsburgh, PA).

The participants were first familiarized with the task in a practice session with 4 trials; the formal experiment would not start until participants’ accuracy reached 90%.

Each trial started with a 500 ms fixation sign. The first word in a pair was then presented for 500 ms, followed by the second word. Participants were asked to judge whether words in the pair were semantically related by pressing the button D (related) or J (unrelated), as soon as they saw the second word. Once the responses of participants were registered, the second word disappeared from the screen. An average experimental session lasted about 15 min.

#### Data preparation

Participants’ reaction time (RT) and accuracy (ACC) were collected and analyzed. Trials with an incorrect response (4.7% of all trials) were excluded from the RT analysis, and so were trials with excessively fast or slow reactions (RT below 300 ms or above 3,000 ms, 5.0% of all trials). Only trials with excessively fast or slow reactions (RT below 300 ms or above 3,000 ms, 5.0% of all trials) were trimmed in ACC analysis. Table 2 displays the RT and ACC means for all conditions.

**TABLE 2:** Mean reaction time (RT, in ms) and accuracy (ACC, in %) of the two proficiency groups in semantic relatedness judgment.

	More proficient group (*n* = 29)		Less proficient group (*n* = 29)	
		
Condition	ACC	RT	ACC	RT
S+T+(*n* = 10)	93.57 (24.57)	1162 (535)	83.67 (37.04)	1272(560)
S+T−(*n* = 10)	86.35 (34.40)	1150 (515)	82.44 (38.11)	1217(574)
S−T+(*n* = 10)	61.54 (48.75)	1469 (551)	54.27 (49.92)	1567 (591)
S−T−(*n* = 10)	96.98 (17.14)	1275(528)	81.33 (39.04)	1545 (628)

Four conditions are presented: semantic related (S+), semantic unrelated (S−), translation repeated (T+), and translation unrepeated (T−). Standard deviations are given in parentheses.

Data analyses were run using R (R Core Team, 2019). There were two types of models conducted: (a) to investigate the possible effect of semantic-relatedness and translation-repetition, fixed effects in the model were established as: semantic relatedness (related, unrelated), translation repetition (repeated, unrepeated), and L2 proficiency (the higher proficiency group, the lower proficiency group); (b) to investigate the influence of L2 on the possible L1 translation involvement, we conducted models under two different translation repetition conditions, in which the fixed effect was set as L2 proficiency (the higher proficiency group, the lower proficiency group). For both model types, the random effects structure included by-participant and by-item effects.

The above two model types were both associated with an RT, as well as an ACC analysis. In RT analysis, raw data was log-transformed to compensate for the lack of a normal distribution. The models were then conducted as linear mixed-effect ones with the *LmerTest* package (Kuznetsova et al., 2017). Analysis-of-variance was calculated using the function of *anova*. *Post-hoc* group comparisons were made using the *emmeans* package (Lenth et al., 2020). And in ACC analysis, models were conducted as logic mixed-effect ones with the *LmerTest* package. Analysis-of-variance was calculated using the package *CAR* (Fox and Weisberg, 2019). Similarly, *post-hoc* group comparisons were made using the package *emmeans*.

### Results

#### The semantic relatedness effect

There existed the main effect of semantic relatedness in RT [*F*(1) = 22.28, *p* < 0.001], as well as in ACC [χ^2^(1) = 6.39, *p* = 0.011]. *Post-hoc* group comparison demonstrated that, participants exhibited a faster reaction (β = −0.25, SE = 0.05, *t* = −4.68, *p* < 0.001) and higher accuracy (β = 0.70, SE = 0.40, *z* = 1.76, *p* = 0.078) to semantically related word pairs than to unrelated ones. Such a better performance suggested the semantic relatedness effect.

#### The translation repetition effect

Importantly, the main effect for translation repetition was reported in both RT [*F*(1) = 4.39, *p* = 0.043] and ACC analysis [χ^2^(1) = 23.97, *p* < 0.001]. *Post-hoc* group comparison revealed that word pairs with a repeated character in translation, compared to those without, induced a slower reaction (β = 0.10, SE = 0.05, *t* = 2.09, *p* = 0.043) and lower accuracy (β = −0.85, SE = 0.41, *z* = −2.08, *p* = 0.037). Such translation repetition effect showed that the manipulation of L1 translation exerted an influence on participants’ performances, indicating the L1 involvement when processing L2 words.

To further investigate the effect of translation repetition, we focused on the two-way interaction of semantic relatedness and translation repetition, which reached marginal significance in RT [*F*(1) = 3.65, *p* = 0.062] and significance in ACC [χ^2^(1) = 22.17, *p* < 0.001]. Further *post-hoc* analysis showed that on the semantic-unrelated condition, exposure to word pairs with translation overlaps impeded participants’ judgment (shown as slower reaction: β = 0.19, SE = 0.07, *t* = 2.77, *p* = 0.008; and lower accuracy: β = −2.31, SE = 0.57, *z* = −4.05, *p* < 0.001), in comparison to word pairs without the translation overlaps. No such effect was found on the semantic-related condition. This may be due to the over-generalization of participants: without the explicit information given on semantics, an association may be assigned between the semantically unrelated words (i.e., they were wrongly assumed to be semantically related) because their L1 translations are somehow associated. But once words are related in meaning, which explicitly reveals the semantic relatedness, participants can judge easily without any help from such generalized “association.” That is why the translation repetition effect was found only under the condition of semantic-unrelatedness.

To sum up, in the task of semantic relatedness judgment (Experiment 1), only a manipulated character of L1 translation was enough to affect the L2 word processing. It is thus necessary to take into account results of the lexical decision task (Experiment 2), in which the L1 translation word is manipulated as a whole. In such a manner we can examine whether a whole L1 translation word can get involved in L2 word processing.

#### Performances between proficiency groups

The main effect of L2 proficiency was reported in RT [*F*(1) = 8.13, *p* = 0.006] and ACC analysis [χ^2^(1) = 28.00, *p* < 0.001]. Unsurprisingly, the *post-hoc* group comparison provided evidence for a faster response (β = −0.13, SE = 0.04, *t* = −2.85, *p* = 0.006) and higher accuracy (β = 1.02, SE = 0.18, *z* = 5.75, *p* < 0.001) in the more proficient group.

To clarify how the two proficiency groups were affected by the manipulated L1 translation, we conducted models on different conditions of translation repetition, focusing on the effect of L2 proficiency. Under the condition of no translation repetition, the main effect of L2 proficiency was significant [RT: *F*(1) = 11.57, *p* < 0.001; ACC: χ^2^(1) = 24.99, *p* < 0.001). The condition of translation repetition also led to a significant main effect of L2 proficiency [RT: *F*(1) = 12.70, *p* < 0.001; ACC: χ^2^(1) = 10.87, *p* < 0.001]. Further *post-hoc* comparisons revealed that the more proficient group exhibited a quicker reaction and higher accuracy, on the condition of unrepeated translation (RT: β = −0.09, SE = 0.03, *t* = −3.40, *p* = 0.0007, ACC: β = 0.95, SE = 0.20, *z* = 4.84, *p* < 0.001) and repeated translation (RT: β = −0.11, SE = 0.03, *t* = −3.56, *p* = 0.0004; ACC: β = 0.48, SE = 0.15, *z* = 3.28, *p* = 0.001). In sum, no contrast was found between the two conditions.

According to the task of semantic relatedness judgment (Experiment 1), it remained unclear which proficiency group gained greater influence of the involved L1 translation word. That brings the necessity to check the results of the lexical decision task (Experiment 2).

## Experiment 2: Lexical decision

### Method

#### Participants

Same as Experiment 1.

#### Materials

The experiment follows the materials by Jiang et al. (2019). Our test materials consisted of 64 English words (the target stimuli), 48 non-words and 16 English filler words. All of the materials were randomly presented.

Among the target stimuli, there were 32 words matched for length and lexical frequency, but differed in translation frequency (the frequency of its Chinese translation): half of them were with relatively high-frequency Chinese translations (HTF, high translation frequency), and the other half were with low-frequency ones (LTF, low translation frequency). Another 32 words, matched for length and translation frequency, were differed in lexical frequency: half of them were relatively high-frequency words (HLF, high lexical frequency) while the other half were low-frequency ones (LLF, low lexical frequency) (see Table 3).

**TABLE 3:** Conditions (the mean frequency, length, and translation frequency of target words) in the lexical decision task.

Condition	Target words (Examples)	Frequency (Per million)	Length (Number of letters)	Chinese translations	Translation frequency (Per million)
HTF	Research	22.1	7.5	研究	810.3
LTF	Evidence	23.9	7.5	证据	27.8
HLF	Room	307.8	6.3	房间	76.3
LLF	Carpet	21.6	6.6	地毯	77.8

(1) Target words were all nouns or verbs, each had a unique disyllabic Chinese translation. According to Jiang et al. (2019), the high/low frequency of the English word was based on Brysbaert and New (2009); that of the Chinese word was based on Beijing Language Institute [BLI], 1986. (2) The factors of Frequency and Translation Frequency were not correlated: (r = −0.181, *p* = 0.152).

#### Procedure

Participants were tested individually with their informed consent. Instructions of the lexical decision task were presented to participants orally and visually, with an emphasis on both speed and accuracy. Stimuli of the decision task were all presented visually, using the E-Prime 2.0 software (Psychology Software Tools, Pittsburgh, PA).

The participants were first familiarized with the task in a practice session with 10 trials; the formal experiment would not start until participants’ accuracy reached 90%.

Each trial began with a fixation sign, lasting for 500 ms. Thereafter the letter string was presented at the center of the screen, remaining until participants made the decision. Participants decided on whether the string made up a word or not, by pressing the button D (yes) or J (no). An average experimental session lasted about 15 min.

#### Data preparation

Participants’ reaction time (RT) and accuracy (ACC) were collected and analyzed. In RT analysis, trials with an incorrect response (4.7% of all trials), as well as those with excessively fast or slow reactions (RT below 300 ms or above 3,000 ms, 5.0% of all trials) were excluded. In ACC analysis, only trials with excessively fast or slow reactions (RT below 300 ms or above 3,000 ms, 5.0% of all trials) were trimmed. Table 4 displays the RT and ACC means for all conditions.

**TABLE 4:** Mean reaction time (RT, in ms) and accuracy (ACC, in %) of the two proficiency groups in lexical decision task.

	More proficient group (*n* = 29)		Less proficient group (*n* = 29)	
		
Condition	ACC	RT	ACC	RT
HLF (*n* = 16)	99.12 (9.33)	895 (401)	97.16(16.64)	972 (458)
LLF (*n* = 16)	97.98 (14.09)	1,015 (471)	91.80 (27.46)	1,091 (518)
HTF (*n* = 16)	96.20 (19.15)	1,057 (443)	93.02 (25.51)	1,167 (540)
LTF (*n* = 16)	98.86 (10.64)	1,151 (520)	89.86 (30.22)	1,211 (561)

Four word conditions are presented as words with high frequency (HLF), low frequency (LLF), high translation frequency (HTF), and low translation frequency (LTF). Standard deviations are given in parentheses.

In the present experiment, the same methods and packages for data analysis in Experiment 1 were adopted. Differed from Experiment 1, though, three types of models were conducted: (a) to verify the effect of lexical frequency, we focused on target words with LLF and HLF, setting the fixed effects as lexical frequency (high, low) and L2 proficiency (the higher proficiency group, the lower proficiency group); (b) to explore the possible effect of translation frequency, we chose the target words with LTF and HTF, setting the fixed effects as translation frequency (high, low) and L2 proficiency (the higher proficiency group, the lower proficiency group); (c) to investigate the influence of L2 on the possible L1 translation involvement, we conducted models on two different translation frequency conditions, in which the fixed effect was set as L2 proficiency (the higher proficiency group, the lower proficiency group). For each of the three types of models, the random effects structure included by-participant and by-item effects.

### Results

#### The lexical frequency effect

A main effect of lexical frequency was revealed in RT [*F*(1) = 33.11, *p* < 0.001]. It can be seen from the *post-hoc* comparison that, the words of higher frequency led to participants’ quicker reactions compared to the words of lower frequency (β = −0.11, SE = 0.02, *t* = −5.82, *p* < 0.001), known as the lexical frequency effect. However, no significant main effect of lexical frequency was reported in ACC.

#### The translation frequency effect

To be noted, RT analysis yielded a main effect for translation frequency [*F*(1) = 7.42, *p* = 0.007]. *Post-hoc* group comparison revealed that participants responded to higher translation frequency words faster than to lower translation frequency words (β = −0.06, SE = 0.02, *t* = −2.75, *p* = 0.006). It was an influence brought about by the manipulation of L1 translation, i.e., the translation frequency effect, which indicated the L1 involvement in L2 word processing.

The translation-frequency effect was not reported in ACC analysis. Yet a visual inspection of the data in the less proficient group (see Table 4) might indicate an ACC difference in conditions of higher- and lower-translation frequency (the more proficient group, however, who exhibited a rather high ACC, may exhibit a ceiling effect): words with a higher translation frequency induced higher accuracy, compared to those with a lower translation frequency. It indicated an easier processing of words with higher translation frequency, possibly serving as a verification of translation frequency effect in RT analysis.

Further, the translation frequency effect was independent of the lexical frequency effect: in the present experiment, the translation frequency effect was obtained from words matched in their lexical frequency. In this way, a more reliable conclusion can be reached.

#### Performances between proficiency groups

A main effect of L2 proficiency was shown in target words with manipulated lexical frequency [RT: *F*(1) = 12.55, *p* < 0.001; ACC: χ^2^(1) = 5.09, *p* = 0.024], as well as in those with manipulated translation frequency [RT: *F*(1) = 9.92, *p* = 0.002; ACC: χ^2^(1) = 4.48, *p* = 0.034]. *Post-hoc* comparison demonstrated that, the more proficient group exhibited a quicker response and higher accuracy, no matter when given target words varying in lexical frequency (RT: β = −0.07, SE = 0.02, *t* = −3.53, *p* < 0.001; ACC: β = 1.33, SE = 0.35, *z* = 3.86, *p* < 0.001) or in translation frequency (RT: β = −0.06, SE = 0.02, *t* = −3.13, *p* = 0.002; ACC: β = 1.46, SE = 0.29, *z* = 5.13, *p* < 0.001).

Additionally, the effect of L2 proficiency was analyzed separately in two translation frequency conditions, through which we can distinguish how different proficiency groups were affected by the manipulated L1 translation. On condition of lower translation frequency, the main effect of L2 proficiency was reported in only ACC analysis [χ^2^(1) = 37.56, *p* < 0.001]; in *post-hoc* analysis, the higher accuracy in the more proficient group was revealed (β = 2.28, SE = 0.48, *z* = 4.78, *p* < 0.001). However, on condition of the higher translation frequency, the main effect of L2 proficiency was observed in both RT [*F*(1) = 8.70, *p* = 0.003] and ACC [χ^2^(1) = 4.48, *p* = 0.034]; and in the *post-hoc* analysis, the faster response (β = −0.08, SE = 0.03, *t* = −2.95, *p* = 0.003), along with higher accuracy (β = 0.64, SE = 0.31, *z* = 2.07, *p* = 0.038) was obtained in the more proficient group. Overall, a contrast has been revealed: words with lower translation frequency led to nearly the same reaction speed across the two groups; when processing words with higher translation frequency, however, the more proficient group was significantly quicker than its less proficient counterpart. We can tell from the contrast that it was the more proficient participants, not the less proficient ones, who “gained more benefits” from the high-frequent L1 translation. It suggested a greater influence of L1 translation once the bilingual reaches a higher proficiency level.

## General discussion

The present study adopted the tasks of semantic relatedness judgment and lexical decision, testing both the higher- and lower-proficiency bilinguals. Results revealed that participants performed better on semantically related word pairs and higher frequency words. More importantly, participants’ worse performances were observed when the word pair shared a repeated character in L1 translations, or when the target words were with lower-frequency L1 translations, demonstrating that the manipulated L1 translation exerts an influence on bilinguals’ task performances. We also found that the more proficient bilinguals “gained more benefits” from the high-frequency L1 translation, suggesting that the manipulated L1 translation had an even greater influence on the more proficient bilinguals, than their less proficient counterparts. Further discussions on the effects by the manipulated L1 translation, the L2 proficiency and the task demands, are presented as follows.

### The involvement of L1 translation in L2 word processing

The present study aimed to explore the role of L1 translation in L2 word processing. Results showed that the words that share a character in their Chinese translations led to worse performances in the judgment of semantic relatedness (the translation repetition effect); words with a high frequency Chinese translations promoted the lexical decision (the translation frequency effect). The manipulation of not only the form, but the frequency of L1 translation had an effect when bilinguals processed L2 words, which revealed the involvement of L1 translation in L2 word processing.

That is, we suggested an L1 translation mediation, but not a strong and direct association between L2 lexicon and the word meaning. In fact, for the unbalanced bilinguals (as recruited in the present study), the L2 lexicon is learned after the complete construction of the concept (i.e., the word meaning). The connection between the concept and L2 lexicon can thus be weak. Such weak connection is supported by the asymmetric cross-language priming (which is weaker from L2 to L1 than that from L1 to L2, see, e.g., Keatley et al., 1994; Jiang, 1999), as well as the reduced emotional responses in L2 processing (see, e.g., Costa et al., 2014).

The L1 translation involvement has been well documented in a variety of participants and conditions. In the task of semantic relatedness judgment, for example, Thierry and Wu (2004, 2007) revealed an unconscious translation into L1 in proficient bilinguals’ L2 comprehension. Zhang et al. (2012) replicated this pattern of results in late, non-proficient bilinguals. Xiao and Ni (2016) subdivided the condition “character repetition (in the target word’s Chinese translation)” into the “first/final-character repetition,” suggesting a translation into L1 even at the sub-lexical level. And in a lexical decision task, Jiang et al. (2019) recruited bilinguals with immersion experience in L2 and found also the influence of L1 translation, establishing that L1 translation served as an integral part of L2 word processing.

The phenomenon can be discussed in Jiang’s Three-Stage Model of L2 Lexical Development (Jiang, 2000), according to which the developing L2 lexicon consists of three gradual stages: (a) the formal stage when a link between L2 words and L1 translations is established; (b) the L1 lemma mediation stage with a stronger L1–L2 link, when L2 word processing is mediated by lemmas of its L1 translation; (c) the L2 integration stage when L2 word information is represented independently, causing the L1–L2 link to be unnecessary in L2 word processing. The L2 mental lexicon of our participants may have reached the second stage (the intermediate level of L2 proficiency), during which L2 words are processed *via* their L1 translation equivalents. It may explain why any manipulation of the L1 translation (its repetition of form, or its frequency) will exert a significant effect on L2 word processing. And notably, the mental lexicon of our higher-proficiency bilinguals stopped at the same stage (the second stage) as their lower-proficiency counterparts. In other words, their lexical development, to some extent, is fossilized (for similar discussion, see Ma, 2015). And it is the L1 translation involvement that can be a major cause. In L2 word processing, the word meaning is mediated by the L1 translation; bilinguals may get used to the “walking stick” and pay less attention to the semantic context, within which the L2 word meaning can be analyzed and acquired (Mestres-Missé et al., 2007, 2014; Wu and Feng, 2014).

### The influence of L2 proficiency

Additionally, we attempted to clarify how L2 proficiency affects the involvement of L1 translation. In RT analysis of the lexical decision task, two proficiency groups performed no differently on words with lower translation frequency; however, the higher-proficiency group was more sensitive than the lower-proficiency one while responding to words with higher translation frequency. The contrast indicated that participants with higher L2 proficiency appeared to benefit more from the L1 translation in L2 word processing. It suggested a better access to L1 translation as one’s L2 proficiency increases.

The phenomenon can also be discussed within Jiang’s Three-Stage Model of L2 Lexical Development. The stages in the model are not that clear-cut; the representations of bilingual’s mental lexicon are in transition from one stage to another (Jiang, 2000). The model’s first stage is with a weak L1–L2 link, which develops to be strong at the second stage, it is thus possible that the L1–L2 link at the second stage is gaining strength progressively, as the bilingual gets more proficient in L2. On this basis, we believe that although our two proficiency groups are undergoing the same stage of L1 lemma mediation, their L1–L2 link can differ in strength. Compared to the lower-proficiency ones, the higher-proficiency group exhibited a stronger link, making possible their easier access to both the L2 word and its L1 translation.

In our lexical decision task, the higher-proficiency bilinguals showed better access to L1 translation, which facilitated the task behavior. It seemed to be a kind of “advantage.” In some tasks of semantic relatedness judgment, however, the higher-proficiency bilinguals’ better access to L1 translation was found and reported as a “disadvantage”: the higher-proficiency bilinguals exhibited worse performance on the condition of L1 translation repetition, compared with the condition of no repetition (Li et al., 2018; Qu, 2019). The distinction may result from the design of the two tasks. In the task of semantic relatedness judgment, the semantic association can be wrongly established due to the form-repeated L1 translation; the L1 translation involvement behaved as a hinderance. And in the lexical decision task, the word recognition can be improved due to the high-frequency L1 translation; the L1 translation involvement acted as an assistance. We thus propose that it is the task itself, rather than the involved L1 translation, that creates such a “disadvantage” or “advantage.”

But notably, we suggest that the more proficient bilinguals exhibit a better access to L1 translation, which does not mean they rely more on the L1 translation involvement. In fact, the involvement of L1 translation seems to decrease, as one’s L2 proficiency increases. In some studies, for example, no better performances were reported even if the L1 translation could be taken as a clue to promote the lexical decision (Hu and Qi, 2014; Jiang et al., 2019). It is because that differed from the present study, participants of those studies have reached quite a high level of L2 (they were university teachers with overseas education experience, see Hu and Qi, 2014; graduate students and visiting scholars studying at an American university, see Jiang et al., 2019). For those highly proficient bilinguals, their L2 mental lexicon can thus be approaching the stage of L2 integration, which makes the L1–L2 link and the L1 word information unnecessary in the L2 word processing. Therefore, in lexical decision tasks, those highly proficient bilinguals were almost independent of the L1 translation.

### The task demands and the depth of L1 translation involvement

Additionally, it was found in the task of semantic relatedness judgment that, a form-repetition in L1 translation was enough to mediate L2 word processing. As for the lexical decision task, however, it was a whole L1 translation word that mediated the L2 processing. The contrast revealed that the depth of L1 translation involvement would change with the task demand; if a character of L1 translation word is sufficient for a certain L2 task, the involvement of a whole L1 translation word becomes unessential.

Such an effect of task demands has been discussed in the BIA+ model. The model is concerned with the processing of bilingual words. It consists of an identification system, which provides the word representations at the semantic, orthographic (lexical) and phonological levels; and a task schema, which takes a decision on how the response will be made. The task schema tends to optimize one’s performances, based on an internal criterion (e.g., the shortest reaction time and the highest accuracy enough for accomplishing the task, which depends mainly on the task demand) (see Dijkstra and Van Heuven, 2002; Dijkstra, 2005). It seems to be in accord with the cognitive economy principle. And in the present study, the participant was able to “take a shortcut”: they used only a form-repetition of, but not a whole L1 translation to mediate L2 word, through which a quicker reaction became possible. It is in essence an optimization of one’s performances, or a kind of task-modulation, conducted by the task schema.

The optimization may be associated with the independence of task schema. The task schema to some extent functions on its own, separate from the identification system (Dijkstra, 2005). In this view, it is possible that in the semantic relatedness judgment, the representation of a word’s L1 translation is activated, waiting for the possible reaction optimization. The task schema thereafter conducts the reaction optimization, according to which only a form-repetition of the L1 translation will be selected and taken advantage of, so that the L2 word processing can be mediated. That is to say: the whole L1 translation word is activated (which serves as a by-product), while it is only a form-repetition in L1 translation that gets involved in L2 word processing, due to the effect of task demand.

The effect of task demand, according to BIA+ model, is usually depicted on a broader scale. However, we found in the present study that it can work at a narrower range. It is obvious that in the task of semantic relatedness judgment, due to the L1 translation involvement, both the lexical and the semantic level of L1 words are useful to arrive at a response; but as for the lexical decision task, the processing at the semantic level becomes less critical. Thus in differed tasks, the language (L1) involvement may reach different levels (for similar discussion, see Wang et al., 2011). It can be regard as a task-modulation across the language levels, conducted by the task schema. That is the most common interpretation based on the BIA+ model. The present study, however, turned to a more specific scale. In both tasks of the present study, the L1 translation involvement has reached the lexical level. Experiment 2 reported a fully involved L1 translation word at the lexical level, while Experiment 1 suggested an involvement of L1 form-repetition; it is a partially involved L1 translation at the lexical level. The contrast of the two experiments showed that even at a certain language level, the depth of word processing could differ, if the task demands allowed. In other words, the task-modulation (derived from the operation of task schema, according to BIA+ model) may play a role not only across the language levels, but just at a certain level.

## Conclusion

This article investigated the role of L1 translation in L2 word processing, while taking into consideration the influence of L2 proficiency and task demands. Results showed that the performances of participants were affected by the manipulated L1 translation, indicating an involvement of L1 translation in L2 word processing. It was also found that compared with the lower-proficiency ones, the higher-proficiency bilinguals could be more sensitive to the manipulated L1 translation, demonstrating their better access to both the L2 word and its L1 translation. Additionally, the depth of L1 translation involvement was found to vary with the task demands. It suggested a kind of task-modulation at a certain language level, which may provide an uncommon viewpoint in interpreting the BIA+ model.

## Data availability statement

The original contributions presented in this study are included in the article/Supplementary material, further inquiries can be directed to the corresponding author.

## Ethics statement

Ethical review and approval was not required for the study on human participants in accordance with the local legislation and institutional requirements. The patients/participants provided their written informed consent to participate in this study.

## Author contributions

TZ: conceptualization, project administration, writing—review and editing, and supervision. CC: validation, data curation, formal analysis, and writing—original draft. JG: methodology and investigation. All authors contributed to the article and approved the submitted version.
