# Supplementary material for: First language translation involvement in second language word processing
Source: Front Psychol. 2022 Sep 8;13:986450. doi: 10.3389/fpsyg.2022.986450 (PMC9493259; doi:10.3389/fpsyg.2022.986450)
Supplement: Supplementary file 1 [file Data_Sheet_1.pdf]

Appendix: Materials of the experiments

1. Word pairs used in Experiment 1 (semantic relatedness judgment)

Word pairs	Translation	SRE	SRC
(S+T+)			
courage-warrior	勇气-勇士	4.6	4.6
college-student	学院-学生	4.8	4.9
court-law	法院-法律	4.9	5.0
kitchen-cook	厨房-厨师	5.0	5.0
hospital-doctor	医院-医生	5.0	5.0
garden-flower	花园-花朵	4.8	4.9
post-mail	邮政-邮件	4.7	4.6
calendar-date	日历-日期	4.8	4.7
math-number	数学-数字	4.9	4.8
umbrella-raincoat	雨伞-雨衣	4.7	4.6
(S-T+)			
novel-thief	小说-小偷	1.4	1.2
expression-surface	表达-表面	1.2	1.3
ham-flame	火腿-火焰	1.2	1.5
sofa-desert	沙发-沙漠	1.3	1.0
kite-storm	风筝-风暴	1.6	1.7
machine-chance	机器-机会	1.0	1.3
message-trust	信息-信任	1.3	1.4
angel-genius	天使-天才	1.3	1.2
professor-coach	教授-教练	2.2	2.3
space-air	空间-空气	2.4	2.5
(S+T-)			
lamp-desk	台灯-桌子	4.2	4.1
teacher-blackboard	老师-黑板	4.8	4.7
knowledge-theory	知识-理论	4.1	3.9
ill-danger	疾病-危险	4.0	3.6
milk-bread	牛奶-面包	4.6	4.0
opera-actor	歌剧-演员	4.7	4.7
vegetable-lunch	蔬菜-午餐	4.6	3.8
sky-star	天空-星星	4.8	4.5
success-game	成功-游戏	3.6	3.0
science-computer	科学−电脑	4.1	4.0

(S-T-)			
bottle-intelligence	瓶子-智力	1.1	1.2
cake-concept	蛋糕-概念	1.1	1.2
sport-fork	运动-叉子	1.0	1.0
driver-exhibition	司机-展览	1.8	1.4
frequency-cheese	频率-奶酪	1.1	1.1
literature-partner	文学-伙伴	1.4	1.4
tool-grammar	工具-语法	2.1	2.6
forest-adult	森林-成人	1.1	1.2
nose-train	鼻子-火车	1.6	1.2
center-bird	中心-鸟儿	1.1	1.1

Note: 1. The word pairs were divided according to its semantic relatedness (S+: semantic related; S-: semantic unrelated) and repetition in translation (T+: translation repeated; T-: translation unrepeated). 2. The semantic relatedness of the English stimuli was measured by SRE (on a scale of 1 to 5), and the semantic relatedness of the Chinese stimuli was measured by SRC (on a scale of 1 to 5).

2. Target words used in Experiment 2 (lexical decision)

High translation frequency				Low translation frequency			
Target	FE	Translation	FC	Target	FE	Translation	FC
revolution	12	革命	1963	signature	12	签名	3
society	33	社会	1841	contract	33	合同	34
develop	10	发展	1126	appetite	9	胃口	6
enemy	49	敌人	1078	punish	10	惩罚	15
research	33	研究	1001	faith	46	信仰	7
economy	6	经济	989	repeat	33	重复	49
chairman	12	主席	710	attract	6	吸引	51
study	49	学习	679	furniture	15	家具	30
history	84	历史	678	memory	49	记忆	62
politics	17	政治	631	evidence	85	证据	23
relation	4	关系	623	disaster	17	灾难	35
compare	15	比较	417	dictionary	4	词典	5
peasant	4	农民	290	apologize	8	道歉	12
content	8	内容	187	warning	32	警告	21
classmate	2	同学	153	liquid	8	液体	44
technology	15	技术	599	patience	15	耐心	48
High lexical frequency				Low lexical frequency			
Target	FE	Translation	FC	Target	FE	Translation	FC
guess	454	猜测	8	difficulty	6	困难	344
music	152	音乐	54	tuition	4	学费	11
room	440	房间	62	flood	6	水灾	4
husband	195	丈夫	62	hero	50	英雄	148
trouble	224	麻烦	63	allow	32	允许	102
understand	482	理解	77	suggest	39	建议	68
welcome	179	欢迎	109	pollution	2	污染	60
name	642	名字	138	wage	3	工资	49
morning	439	早晨	124	correct	45	纠正	56
remember	542	记得	148	carpet	12	地毯	17
season	187	季节	46	nurse	45	护士	43
church	136	教堂	13	battery	12	电池	18
explain	110	解释	86	scholar	4	学者	26
idea	359	想法	38	describe	17	描写	29
police	236	警察	81	accept	53	接受	126
company	147	公司	111	experiment	16	实验	144

Note: 1. FE refers to frequency of the English target words (per million), while FC refers to frequency of the Chinese translations (per million). 2. Target words of high/low translation frequency were matched for the lexical frequency, while those of high/low lexical frequency were matched for the translation frequency. 3. The materials follows Jiang et al. (2019).
